# Gait Neural Network for Human-Exoskeleton Interaction

**DOI:** 10.3389/fnbot.2020.00058

**Published:** 2020-10-29

**Authors:** Bin Fang, Quan Zhou, Fuchun Sun, Jianhua Shan, Ming Wang, Cheng Xiang, Qin Zhang

**Affiliations:** ^1^Department of Computer Science and Technology, Beijing National Research Center for Information Science and Technology, Tsinghua University, Beijing, China; ^2^Anhui Province Key Laboratory of Special Heavy Load Robot, Anhui University of Technology, Ma'anshan, China; ^3^North Automatic Control Technology Institute, Taiyuan, China; ^4^Department of Physics & Astronomy, Iowa State University, Ames, IA, United States; ^5^State Key Lab of Digital Manufacturing Equipment and Technology, Huazhong University of Science and Technology, Wuhan, China

**Keywords:** exoskeleton, interaction, gait neural network, gait recognition, prediction, temporal convolutional network

## Abstract

Robotic exoskeletons are developed with the aim of enhancing convenience and physical possibilities in daily life. However, at present, these devices lack sufficient synchronization with human movements. To optimize human-exoskeleton interaction, this article proposes a gait recognition and prediction model, called the gait neural network (GNN), which is based on the temporal convolutional network. It consists of an intermediate network, a target network, and a recognition and prediction model. The novel structure of the algorithm can make full use of the historical information from sensors. The performance of the GNN is evaluated based on the publicly available HuGaDB dataset, as well as on data collected by an inertial-based wearable motion capture device. The results show that the proposed approach is highly effective and achieves superior performance compared with existing methods.

## 1. Introduction

The development of lower-extremity robotic exoskeletons (Ackermann and van den Bogert, 2010) has been found to have significant potential in medical rehabilitation (Zhang et al., 2015) and military equipment applications. In these devices, human gait is captured in real time through signals (Casale et al., 2011) that are then sent to a controller. The controller returns instructions to the mechanical device on necessary adjustments or modifications. However, these exoskeletons require more effective prediction modules for joint gait trajectories (Aertbeliën and Schutter, 2014). The main goal is to improve the synchronization between the exoskeleton and human movement (Du et al., 2015). Essentially, the time gap between human action and mechanical device adjustment must be reduced without sacrificing the precision and quality of the modification. To achieve this, it is necessary to mine historical data and understand their intent (Zhu et al., 2016). Time series analysis is a powerful tool for this purpose.

Gait signal collection is commonly performed via inertial measurement units (IMUs), tactile sensors, surface electromyography, electroencephalograms, and so on. The majority of popular gait datasets employ computer vision technology to improve efficiency (Shotton et al., 2011), such as the Carnegie Mellon University Motion of Body dataset (Gross and Shi, 2001), the University of Maryland Human Identification at a Distance dataset, the Chinese Academy of Sciences Institute of Automation Gait Database (Zheng et al., 2011), and the Osaka University Institute of Science and Industry Research Gait Database (Iwama et al., 2012). However, it is often difficult to obtain accurate human gait information from such image-based prediction methods.

The HuGaDB dataset of the University Higher School of Economics contains highly detailed kinematic data for human gait analysis and activity recognition. It is the first public human gait dataset derived from inertial sensors that contains segmented annotations for the study of movement transitions. The data were obtained from 18 participants, and a total of about 10 h of activity was recorded (Chereshnev and Kertesz-Farkas, 2017). However, one limitation of the above-mentioned datasets is that they are not adequate for extremely delicate exoskeleton control.

To achieve sufficiently dexterous and adaptive control, in addition to statistical approximation by Markov modeling, deep learning has been demonstrated to be an effective approach. Recurrent neural networks, such as long short-term memory (LSTM), have been widely used in the areas of time series analysis and natural language processing. The cyclic nature of the human gait has previously precluded the use of such networks (Martinez et al., 2017; Ferrari et al., 2019). Nevertheless, a novel LSTM-based framework has been proposed for predicting the gait stability of elderly users of an intelligent robotic rollator (Chalvatzaki et al., 2018), fusing multimodal RGB-D and laser rangefinder data from non-wearable sensors (Chalvatzaki et al., 2018). An LSTM network has also been used to model gait synchronization of legs using a basic off-the-shelf IMU configuration with six acceleration and rotation parameters (Romero-Hernandez et al., 2019). Further, recent works have reported the use of convolutional neural networks (CNNs) for human activity recognition. CNNs use accelerometer data for real-time human activity recognition and can handle the extraction of both local features and simple statistical features that preserve information about the global form of a time series (Casale et al., 2011). A survey on deep learning for sensor-based activity recognition is presented in Wang et al. (2018).

At present, the majority of gait prediction models are not sufficiently precise or robust with respect to environmental fluctuations. In this work, a gait neural network (GNN) is proposed for gait recognition and prediction through wearable devices. The data-processing component consists of two phases: handling buffer data through an intermediate network and target prediction. Experiments are performed on a public human gait dataset, and the results obtained from the GNN are longitudinally compared with those of other methods. Further, more meticulous gait signals are collected from the IMU to improve training convergence and accuracy. Our model should also be helpful to exoskeletons with inertial sensors.

## 2. Materials and Methods

CNNs are often used for two-dimensional data-processing tasks, such as image classification and target detection. Recently, researchers have found that CNNs can be used to process one-dimensional time series data, where both the convolution kernel and the pooling window are changed from having two dimensions to having just one dimension. In 2018, a temporal convolutional network (TCN) architecture was proposed (Bai et al., 2018). This architecture was deliberately kept simple, combining some of the best practices of modern convolutional architectures. When compared with canonical recurrent architectures, such as LSTM and gated recurrent units, the TCN can convincingly outperform baseline recurrent architectures across a broad range of sequence-modeling tasks. Some scholars have used TCNs in human action segmentation with video or image data (Lea et al., 2016a,b) and medical time series classification (Lin et al., 2019). The distinguishing characteristics of TCNs are that the convolutions in the architecture are causal, meaning that there is no information “leakage” from future to past, and that the architecture can take a sequence of any length and map it to an output sequence of the same length, just as with a recurrent neural network. Therefore, the TCN is used as the base model to handle sequence-modeling tasks, such as obtaining inertial data on human gaits.

### 2.1. Standard Temporal Convolutional Networks

Consider an input sequence *x*_0_, …, *x*_*T*_ for which an output prediction, such as *y*_0_, …, *y*_*T*_ is desired for each time, where *y*_*T*_ depends only on *x*_0_, …, *x*_*T*_, with no future inputs *x*_*t*+1_, …, *x*_*T*_. The sequence-modeling network is any function *f*:*x*^*T*+1^→*y*^*T*+1^ that produces the mapping

(1) $$\begin{array}{r} {\hat{y}}_{0},\ldots ,{\hat{y}}_{T}=f\left(x_{0},\ldots ,x_{T}\right). \end{array}$$

**Figure 1:**
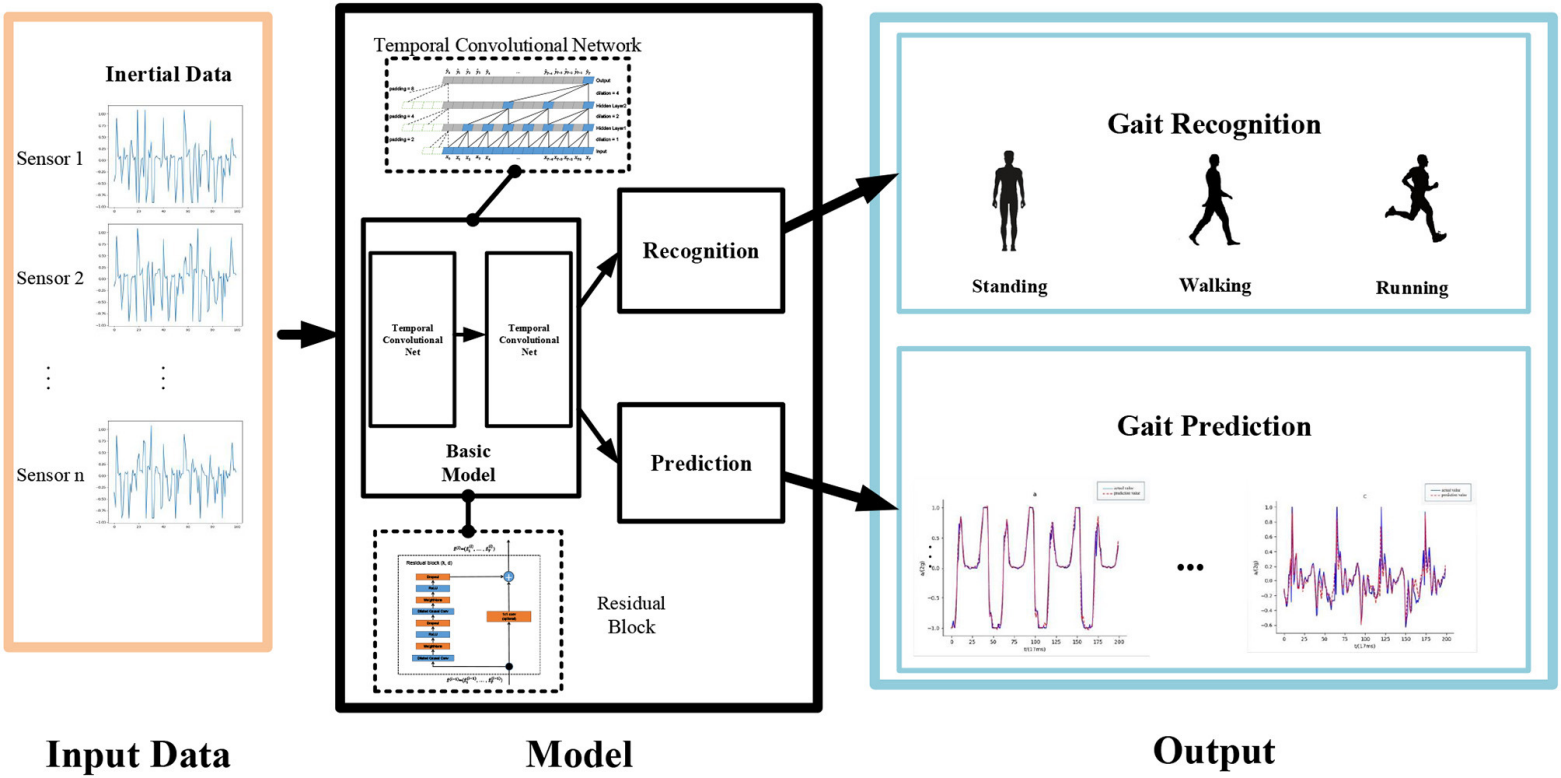
Structure of the GNN.

This sequence-learning algorithm seeks a network *f* that minimizes the loss *L*(*y*_0_, …, *y*_*T*_, *f*(*x*_0_, …, *x*_*T*_)), which measures the difference between the predictions and the actual targets. The TCN employs dilated convolutions to allow an exponentially large receptive field. For a one-dimensional sequence input *x* ∈ ℝ^*n*^ and a filter *f*:{0, …, *k* − 1} → ℝ, the dilated convolution operation on an element of the sequence is defined as

(2) $$\begin{array}{r} F\left(s\right)=\overset{k-1}{\underset{i=0}{\sum }}f\left(i\right)\cdot x_{s-d\cdot i}, \end{array}$$

where *d* is the dilation factor, *k* is the filter size, and *s* − *d*·*i* accounts for the past direction. Thus, dilation is equivalent to introducing a fixed step between every two adjacent filter taps. When *d* = 1, a dilated convolution reduces to a regular convolution. By using a larger dilation, the outputs in the top layer can have a larger receptive field and so represent a wider range of inputs. The basic architectural elements of a TCN are shown in Figure 1.

The output *o* of a layer is related to the input via an activation function:

(3) $$\begin{array}{r} o=\text{Activation}\left(x+\Gamma \left(x\right)\right). \end{array}$$

Within a residual block, the base model has two layers of dilated causal convolution and non-linearity. For the non-linearity, a rectified linear unit is used as the activation function. For normalization, weight normalization is applied to the convolutional filters. For regularization, a spatial dropout is added after each dilated convolution. In a standard residual network the input is directly added to the output of the residual function, whereas in a TCN the input and output can have different widths. To account for possibly different input and output widths, an additional 1 × 1 convolution is used, which ensures that the elementwise addition receives tensors of the same shape.

For convenience, the GNN predicts only the acceleration and gyro data.

Gait prediction is a type of time series prediction. Because it has been shown that TCNs can be very effective sequence models for sequence data (MatthewDavies and Bock, 2019), a TCN is used for human gait analysis in the present work.

A TCN has two characteristics: dilated convolution and causal convolution. The primary function of the dilated convolution is to enable the network to learn more information in a long time series. However, it has been observed that long time series information does not significantly improve the accuracy of gait prediction, because human gait data are periodic and excess information is sampled repeatedly. Therefore, we disregard dilated convolution in this study.

### 2.2. Gait Neural Network

The architecture of the GNN is shown in Figure 1.

**Figure 2:**
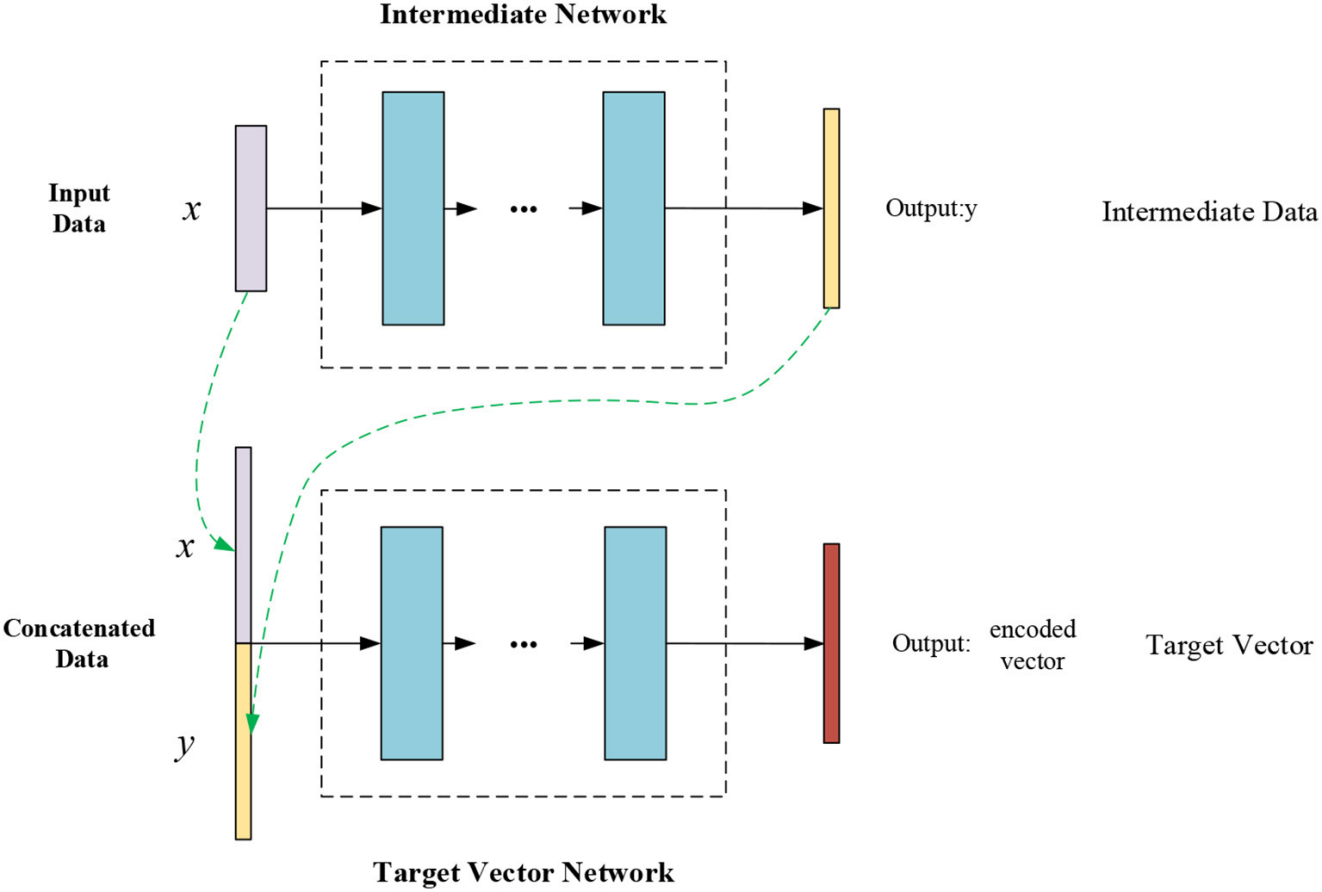
Composition of the basic GNN model.

**Figure 3:**
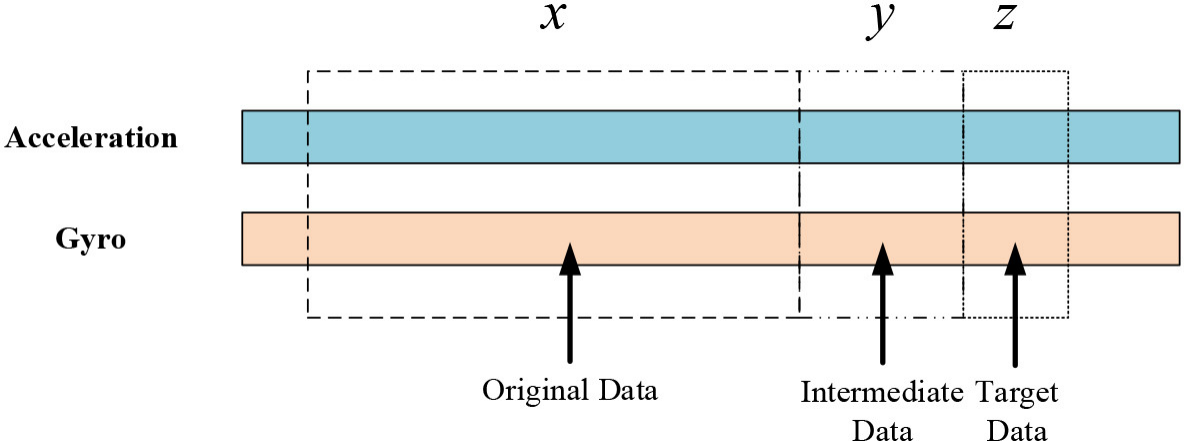
Input data of the GNN.

Two TCNs are used for the basic model. The first is the intermediate network, which uses the normalized inertial data as input to predict the intermediate sensor data. Unlike traditional methods, because of the response delay in the system, the GNN reserves some buffer time, and the original, buffer, and target data are represented, respectively by *x*, *y*, and *z*. Traditional methods generally use *x* to predict *z*, and *y* remains unused. As shown in Figures 2, 3, the GNN uses the original signals as the intermediate network input *x* to predict the intermediate data *y*. The second TCN in the model is the target vector network. The original data *x* and the predicted data *y* are concatenated to make the input to the target vector network, which then outputs the encoded vectors.

Firstly, one TCN is used to process the original input, and then the original input and the output of the first TCN are combined into the input to the second TCN. To a certain extent, it can be seen that the number of features of model learning is increased and the ability to obtain historical information is enhanced.

Finally, a recognition model and a prediction model are added to the network as two fully connected layers. The encoded vectors obtained from the basic model are fed into these recognition and prediction models to output the human action (walking, standing, or running) and the predicted gait data *z* (which mainly includes acceleration and gyro data).

#### 2.2.1. Loss Function

A loss function is used to evaluate the fitting effect of a deep neural network. It is also used to compute gradients using a back-propagation algorithm to optimize the parameters of the network. The GNN has two loss functions to calculate: one for measuring the prediction loss due to prediction error and the other for measuring the loss of recognition accuracy.

The prediction loss function of the GNN is

(4) $$\begin{array}{r} L_{\text{pred}}=w_{y}*L_{y}\left(\hat{y},y\right)+w_{z}*L_{z}\left(\hat{z},z\right), \end{array}$$

where *L*_*y*_ and *L*_*z*_ represent the loss functions of the intermediate prediction network and the target vector network, respectively; *ẑ* and *z* are the output vector and the target vector, respectively; and *w*_*y*_ and *w*_*z*_ are the weight coefficients of *L*_*y*_ and *L*_*z*_, respectively, for which either *L*_1_ or *L*_2_ loss functions can be used:

(5) $$\begin{array}{r} L_{1}=\frac{1}{N_{B}}\overset{N_{B}}{\underset{k=1}{\sum }}\underset{j}{\sum }|{\hat{u}}_{k}^{j}-u_{k}^{j}|, \end{array}$$

(6) $$\begin{array}{r} L_{2}=\frac{1}{2N_{B}}\overset{N_{B}}{\underset{k=1}{\sum }}\underset{j}{\sum }{\left({\hat{u}}_{k}^{j}-u_{k}^{j}\right)}^{2}, \end{array}$$

where *N*_*B*_ denotes the batch size, which is in the range of {32, 64, 128, 256}, *û* is the predicted value of the network output, *u* is the tag value of the network output, and *j* indicates the *j*th output value of the network.

The recognition loss function of the GNN represents the cross-entropy loss:

(7) $$\begin{array}{r} L_{\text{rec}}=-\frac{1}{n}\sum \left[y\ln\hat{y}+\left(1-y\right)\ln\left(1-\hat{y}\right)\right], \end{array}$$

where *ŷ* is the model output.

The total loss of the GNN is

(8) $$\begin{array}{r} L_{\text{total}}=L_{\text{pred}}+\alpha L_{\text{rec}}, \end{array}$$

where α is the hyperparameter used to balance the loss function in order to achieve high performance during the recognition and prediction tasks.

### 2.3. Experimental Approach

In this study, the performance of the GNN was evaluated on two datasets: the publicly available HuGaDB dataset and a human gait dataset obtained using an inertial-based wearable motion capture device.

The GNN was trained by an Adam optimizer with a learning rate of 0.001 at 80 and 150 epochs, divided by 10. The maximum epoch and batch size were 200 and 64, respectively. The dropout rate of all dropout layers was set to 0.3. The GNN was implemented by PyTorch and trained and tested on a computer with an Intel Core i7-8750H processor, two 8 GB memory chips (DDR4), and a GPU (GeForce GTX 1060 6G).

### 2.4. Experiment on a Public Dataset

#### 2.4.1. Gait Data Description

The human body has more than 200 bones. To simplify the gait analysis process, motion analysis is often performed on collected joint motion data. Gait analysis is one method used to study an individual's walking pattern. It aims to reveal the key links and factors influencing an abnormal gait through biomechanics and kinematics, in order to aid clinical diagnosis, guide rehabilitation evaluation and treatment, evaluate efficacy, and inform research on the mechanisms involved. In gait analysis, special parameters are used to assess whether the gait is normal; these generally include gait cycle, kinematic, dynamic, myoelectric activity, and energy metabolism parameters. To improve the correspondence between the robotic exoskeleton and the human body, motion data, such as joint velocity and acceleration data must be collected.

**Figure 4:**
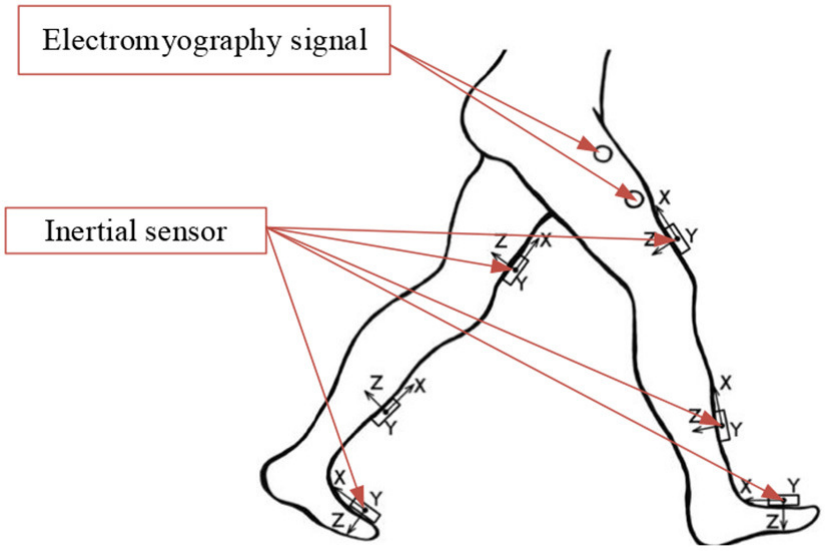
Location of the sensors on the human body that collected data for HuGaDB.

Gait prediction in lower-extremity exoskeleton robots requires highly accurate human gait data, so it is necessary to utilize a gait dataset suitable for human gait prediction. Therefore, we chose to evaluate the gait prediction and recognition performance of the GNN on the publicly available HuGaDB dataset (Chereshnev and Kertesz-Farkas, 2017), which contains detailed kinematic data for analyzing human activity patterns, such as walking, running, taking the stairs up and down, and sitting down. The recorded data are segmented and annotated. They were obtained from a body sensor network comprising six wearable inertial sensors for collecting gait data. Sensors were placed on the left and right thighs, lower legs, and insteps of the human body; their distribution is shown in Figure 4. Each inertial sensor was used to collect three-axis accelerometer, three-axis gyroscope, and occasionally electromyography signals of the corresponding body part, providing data that can be used to evaluate the posture and joint angle of the lower limbs. The data were recorded from 18 participants, and consist of 598 min and 2,111,962 samples in total. The microcontroller collected 56.3500 samples per second on average, with a standard deviation of 3.2057, and transmitted them to a laptop through a Bluetooth connection (Chereshnev and Kertesz-Farkas, 2017). Only the inertial data were taken as input to the present model.

#### 2.4.2. Data Analysis

**Figure 5:**
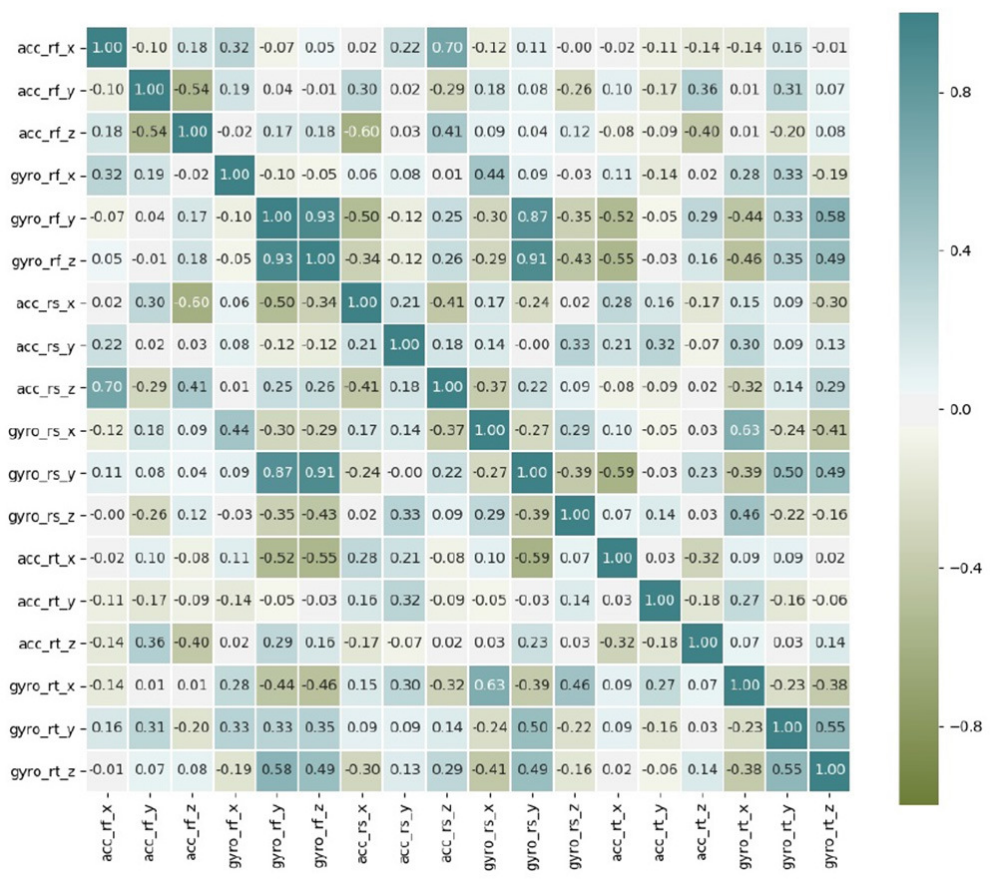
Correlation between the HuGaDB sensor data.

**Figure 6:**
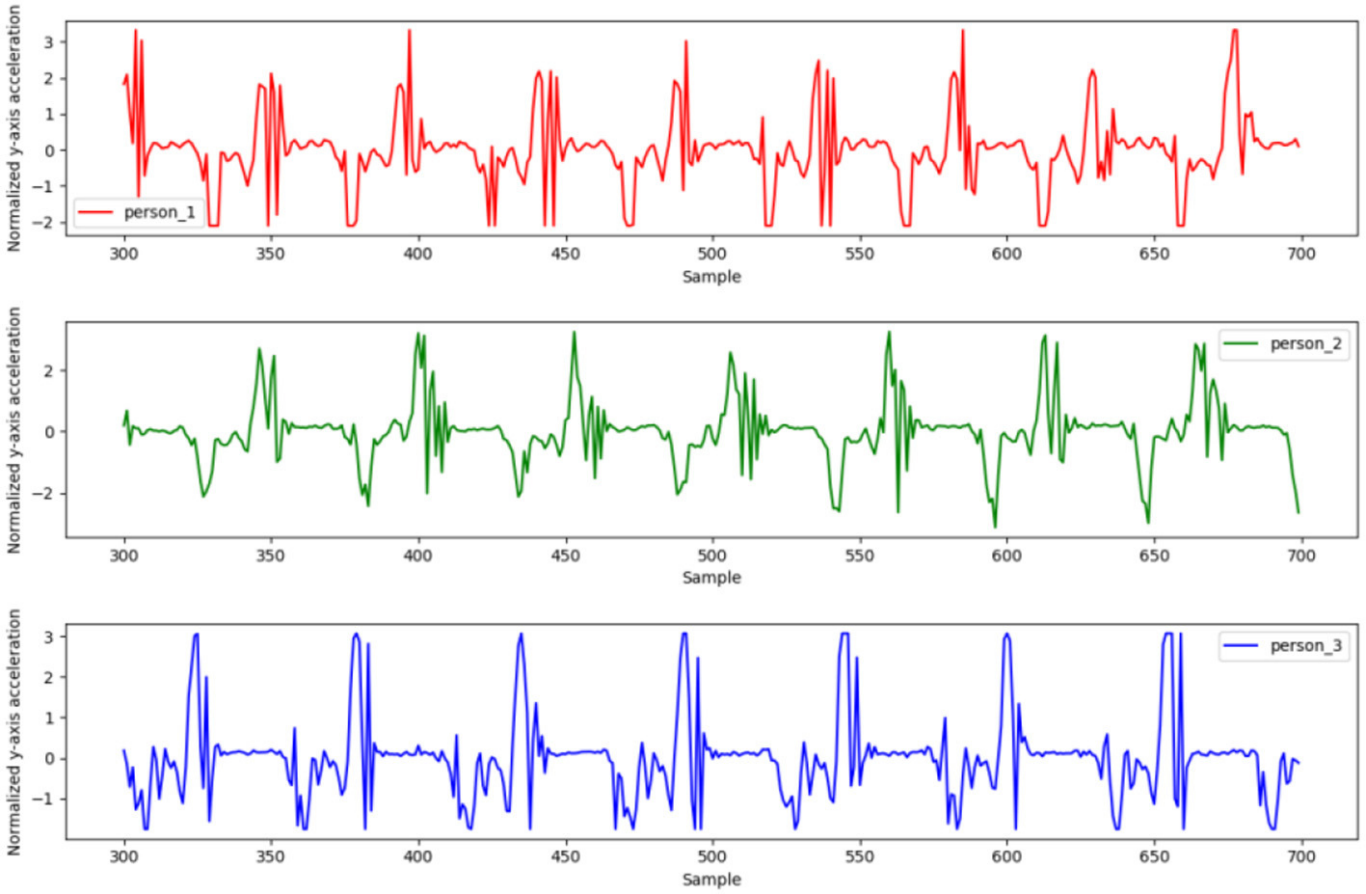
Similarity in the acceleration data of three people.

In this work we analyze gait data from wearable sensors. Given the similarity between the two legs, we use only the right leg as an example. As seen in Figure 5, there is a certain relationship between the sensor signals; hence it is preferable to conduct training using all of the data as input rather than some subset. The acceleration signals of three people were randomly sampled for data analysis. As shown in Figure 6, the gait data are periodic, and the patterns of the three people are similar, although the different individuals have different walking gaits.

#### 2.4.3. Data Pre-processing

The original data in HuGaDB can be converted to values of the corresponding variables. To properly utilize the variables in the GNN, data normalization is necessary. The normalization formula is

(9) $$\begin{array}{r} x_{\text{norm}}=\frac{x-\text{mean}\left(x\right)}{\max\left(x\right)}. \end{array}$$

By this formula, all gait data can be scaled to be between −1 and 1, which can eliminate learning difficulties caused by inconsistencies in data size and range.

#### 2.4.4. Sample Creation

After preprocessing the gait data, the samples used for network training were created. The gait data consist of the acceleration and angular velocity of the inertial devices. In the experiment we conducted, the lengths of *x*, *y*, and *z* were 10, 5, and 1, respectively. To confirm the method of sample creation, samples were selected from the HuGaDB gait data. The first 80% of samples were used as the training dataset, the next 10% were selected for the validation dataset, and the last 10% were taken to be the test dataset.

### 2.5. Experiment on the Collected Data

**Figure 7:**
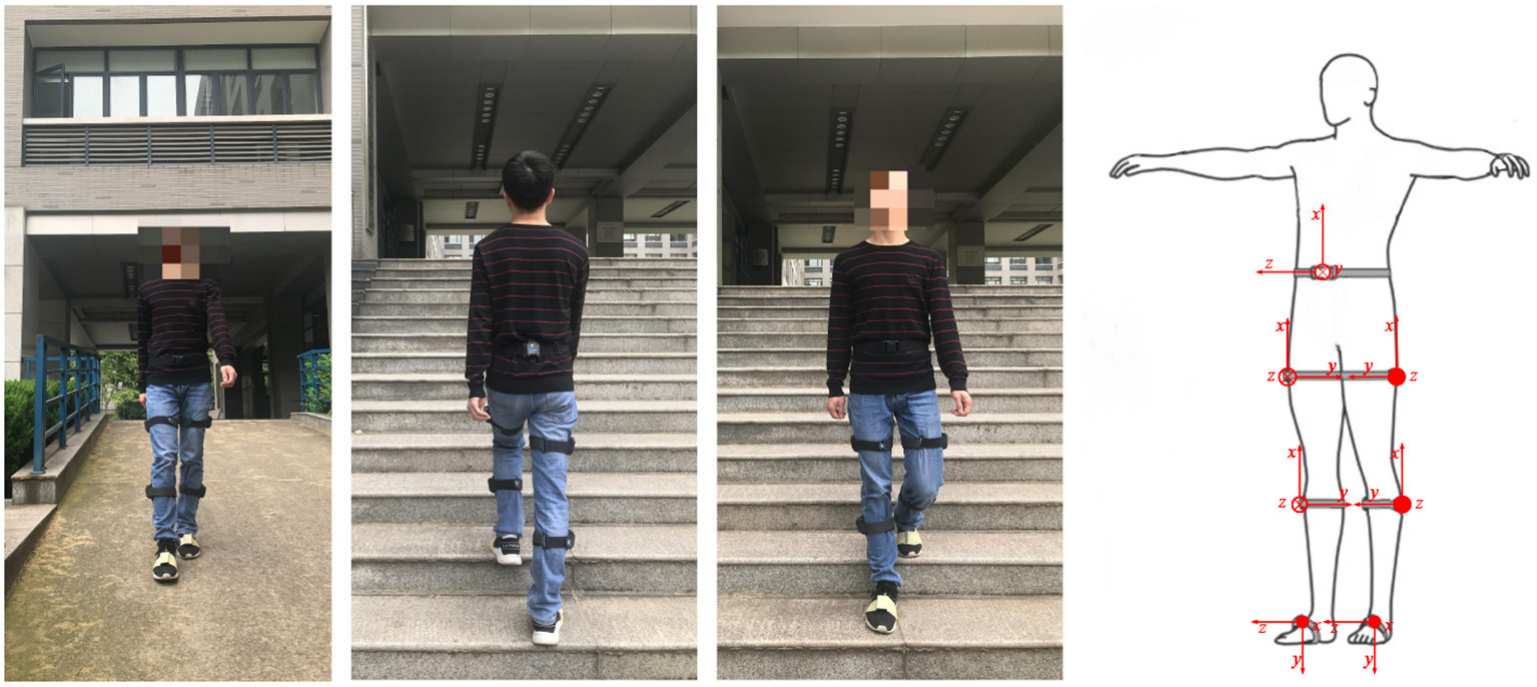
Collected human gait data.

To further evaluate the GNN, an inertial-based wearable motion capture device was used to collect human gait data. The entire motion acquisition system consists of seven inertial measurement units, and only the signals from the lower limbs were selected for the human gait prediction, as shown in Figure 7. Each unit measured the three-axis acceleration and the three-axis angular velocity. The sampling frequency was 120 Hz. The collected gait data include data on walking, going up and down stairs, and going up and down slopes.

## 3. Results

As the predicted data can be collected by inertial sensors, the model is used to predict the accelerations and angular velocities. In the training of the model, multimodal data are used, and the numerical distributions in different dimensions are different; but since we need to use these data as input at the same time, we normalize the data in each dimension. The input and output of the model are multidimensional data, and the units in different dimensions are different. The training of the model is based on the normalized data, and the predicted value of the model is also the accurate value after normalization. Therefore, the mean squared error (MSE) and mean absolute error (MAE) in the evaluation indexes are based on the normalized data, so the units are not specified.

### 3.1. Evaluation Results Using HuGaDB

The network was first trained using the gait data from a single wearer, and the results were compared with those obtained from existing methods. Considering that the exoskeleton must adapt to the movement of different wearers, to test the network's generalization ability, the gait data of three wearers were selected for the training set, and the data of one wearer who was not included in the training set were used for the test set. It took 2.405 s to complete the recognition and prediction task on 2,249 samples in the test set, which means that the prediction and recognition task on each sample took only 1 ms.

#### 3.1.1. Gait Prediction

**Figure 8:**
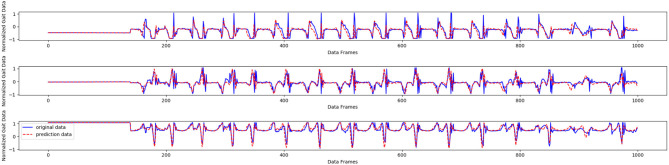
Gait prediction by the GNN based on one-wearer gait data.

The results in Table 1 show that the GNN achieves the best results in the prediction task, with the exception of the maximum error for one wearer. Compared with the other methods, the GNN has the best generalization ability for new human gait data. Further, when the hyperparameter α is set to 0.4 in the experiments, the GNN shows the top performance. As displayed in Figure 8, the gait data of one wearer, including *x*-, *y*-, and *z*-axis acceleration data, were selected to make a prediction; the horizontal parts of the curve represent standing posture, while the oscillating parts represent the walking and running states. We find that the GNN produced good results.

**Table 1:** Comparison of prediction results of different methods on the HuGaDB data.

	**Prediction task**							
			**One-wearer test**			**Generalization test**		
**Method**	**Learning rate**	**Epochs**	**MAE**	**MSE**	**Max error**	**MAE**	**MSE**	**Max error**
**GNN** (α = 0.4)	0.001	200	**0.0427**	**0.0144**	2.06	**0.091**	**0.0277**	**1.760**
LSTM	0.001	200	0.0428	0.01443	2.039	0.1001	0.035	2.126
CNN	0.001	200	0.055	0.0162	1.989	0.1424	0.0474	1.978
BP	0.001	200	0.0522	0.018	2.156	0.1159	0.0406	2.150

*Bold values represent the best performance*.

#### 3.1.2. Gait Recognition

As shown in Table 2, for the single wearer's motion data, all methods achieved good recognition results. When the GNN that was trained on three wearers' data received a new wearer's gait data as input, although it did not achieve the best performance, it did yield a promising accuracy rate of 98.04%.

**Table 2:** Comparison of recognition results of different methods on the HuGaDB data.

**Methods**	**One-wearer test**	**Generalization test**
	**accuracy (%)**	**accuracy (%)**
**RECOGNITION TASK**		
**GNN** (α = 0.4)	**100**	98.04
LSTM	95.67	92.78
BP	97.5	78.49
CNN	96.39	79.24
LightGBM	99.76	**99.33**
SVM	**100**	98.62

*Bold values represent the best performance*.

Based on its performance in the human gait prediction and recognition tasks, we can conclude that the GNN is highly effective in the analysis of human motion.

### 3.2. Evaluation Results Using the Collected Data

To ensure the reliability and fairness of the experiment, all parameters of the model are the same as those used for the HuGaDB dataset.

#### 3.2.1. Gait Prediction

**Figure 9:**
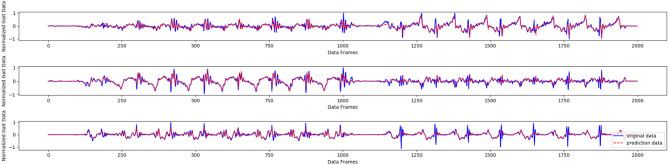
Acceleration prediction result using left tibia data collected from one wearer.

As shown in Figure 9, the acceleration signal of the left tibia was selected as the prediction object. It can be seen that the GNN achieves good prediction performance, except for some abnormal points, which could be caused by noise. It took 1.2332 s to complete the recognition and prediction task on 674 samples in the test set, which means that the prediction and recognition task on each sample took only 1.8 ms.

#### 3.2.2. Gait Recognition

The GNN was compared with other methods on the collected dataset; the results are shown in Tables 3, 4. Clearly, the GNN achieved the best results in most tasks. Further, the MSE value is observed to be larger than the MAE value in the prediction task, as shown in Table 3, indicating that some extreme outliers occur in the data. Again, the GNN achieved superior results in this case.

**Table 3:** Comparison of the prediction results of different methods using the collected data.

**Methods**	**Learning rate**	**Epochs**	**MAE**	**MSE**	**Max error**
**PREDICTION TASK**					
**GNN**	0.001	200	**0.1269**	**0.1314**	**7.28**
LSTM	0.001	200	0.1647	0.2885	9.56
CNN	0.001	200	0.3308	1.4429	15.28
BP	0.001	200	0.1290	0.1391	7.77

*Bold values represent the best performance*.

**Table 4:** Comparison of the recognition results of different methods using the collected data.

**Methods**	**Accuracy (%)**
**RECOGNITION TASK**	
**GNN**	**98.81**
LightGBM	98.34
SVM	97.62
BP	91.68
LSTM	88.78
CNN	85.38

*Bold values represent the best performance*.

Through evaluation on collected datasets, we have verified the feasibility of the model under different data settings. After the model has been trained, it can be deployed on the relevant equipment to achieve real-time and online gait prediction and recognition.

## 4. Conclusions

This article has proposed the GNN as a model for human-exoskeleton interaction. Comparisons of the GNN and other methods on the HuGaDB dataset show that the GNN consistently achieves superior performance. The results further demonstrate that the GNN's generalization performance is better than that of the other methods, despite the increase in the MAE and MSE. Because of the size of the dataset, only three wearers' gait data were used to test the generalization ability. Including more gait data from different groups to train the network should enable even better prediction results to be obtained. For further evaluation of the method, gait data on complex movements were collected using an inertial-based motion capture device. By evaluating the GNN on the collected data, we find that it achieves efficient human gait prediction performance even without strong periodicity. Generally the GNN takes <2 ms to complete the task of gait recognition and prediction. Based on these results, it can be concluded that the GNN model can effectively recognize and predict human motion states.

## Data Availability Statement

The raw data supporting the conclusions of this article will be made available by the authors, without undue reservation, to any qualified researcher.

## Author Contributions

BF designed the network and wrote the manuscript. QZho and JS wrote the code and analyzed the results. FS, MW, and CX helped to improve the manuscript. QZha provided the real experimental data. All authors contributed to the article and approved the submitted version.

## Conflict of Interest

The authors declare that the research was conducted in the absence of any commercial or financial relationships that could be construed as a potential conflict of interest.
